# Solid-State Nuclear Magnetic Resonance as a Tool to Probe the Impact of Mechanical Preprocessing on the Structure and Arrangement of Plant Cell Wall Polymers

**DOI:** 10.3389/fpls.2021.766506

**Published:** 2022-01-12

**Authors:** Coyla R. Munson, Yu Gao, Jenny C. Mortimer, Dylan T. Murray

**Affiliations:** ^1^Department of Chemistry, University of California, Davis, Davis, CA, United States; ^2^Joint BioEnergy Institute, Emeryville, CA, United States; ^3^Environmental Genomics and Systems Biology Division, Lawrence Berkeley National Laboratory, Berkeley, CA, United States; ^4^School of Agriculture, Food and Wine, Waite Research Institute, University of Adelaide, Glen Osmond, SA, Australia

**Keywords:** solid-state nuclear magnetic resonance, *Sorghum bicolor*, cellulose, hemicellulose, lignin, ball milling, recalcitrance (saccharification), preprocessing

## Abstract

Efficient separation of the plant cell wall polymers during lignocellulose processing has been historically challenging due to insolubility of the polymers and their propensity for recalcitrant reassembly. Methods, such as “lignin first” extraction techniques, have advanced efficient biomass use, but the molecular mechanisms for recalcitrance remain enigmatic. Here, we discuss how solid-state Nuclear Magnetic Resonance (NMR) approaches report on the 3D organization of cellulose, xylan, and lignin in the plant cell wall. Recent results illustrate that the organization of these polymers varies across biomass sources and sample preparation methods, with even minimal physical processing causing significant effects. These structural differences contribute to variable extraction efficiencies for bioproducts after downstream processing. We propose that solid-state NMR methods can be applied to follow biomass processing, providing an understanding of the polymer rearrangements that can lead to poor yields for the desired bioproducts. The utility of the technique is illustrated for mechanical processing using lab-scale vibratory ball milling of *Sorghum bicolor*.

## Introduction

Plant lignocellulosic biomass is a sustainable, renewable feedstock for producing bio-based fuels, chemicals, and materials (U.S. Department of Energy, 2016; Li and Takkellapati, 2018). There are two types of plant cell walls: the thin and expandable primary cell wall surrounding all cells and the thicker secondary cell wall deposited at the cessation of cell expansion in some cell types. Due to its thickness, the secondary cell wall forms the vast majority of lignocellulosic biomass (Marriott et al., 2016). It is predominantly composed of the polysaccharide cellulose, a class of polysaccharides called hemicelluloses, and the aromatic polymer, lignin (Marriot et al., 2016). These polymers can then be converted *via* chemical or biological routes into fuels and other valuable chemicals that are conventionally derived from fossil resources (van Putten et al., 2013; Li and Takkellapati, 2018; Baral et al., 2019; Genuino et al., 2019; Priharto et al., 2020).

A major bottleneck in the extraction of chemical precursors from lignocellulosic biomass is the high recalcitrance of the plant cell wall (Marriott et al., 2016; Gilna et al., 2017). Strategies to decrease this intrinsic recalcitrance include the development of plants with altered biomass composition (Pauly and Keegstra, 2010) and novel deconstruction methods, such as the use of new solvents (Kim et al., 2018) and enzyme cocktails (Lopes et al., 2018), which target key polymer linkages. Deconstruction methods can also introduce recalcitrance, for example, by the deposition of more condensed lignin following solubilization (Li et al., 2016). Efforts to reduce recalcitrance include pretreating biomass before deconstruction (Yao et al., 2018; Baig et al., 2019) and “lignin first” deconstruction (Li et al., 2016; Li and Takkellapati, 2018). However, an understanding of how mechanical processing can cause biomass recalcitrance is needed (Gilna et al., 2017; Li and Takkellapati, 2018; Baig et al., 2019).

**Figure 1:**
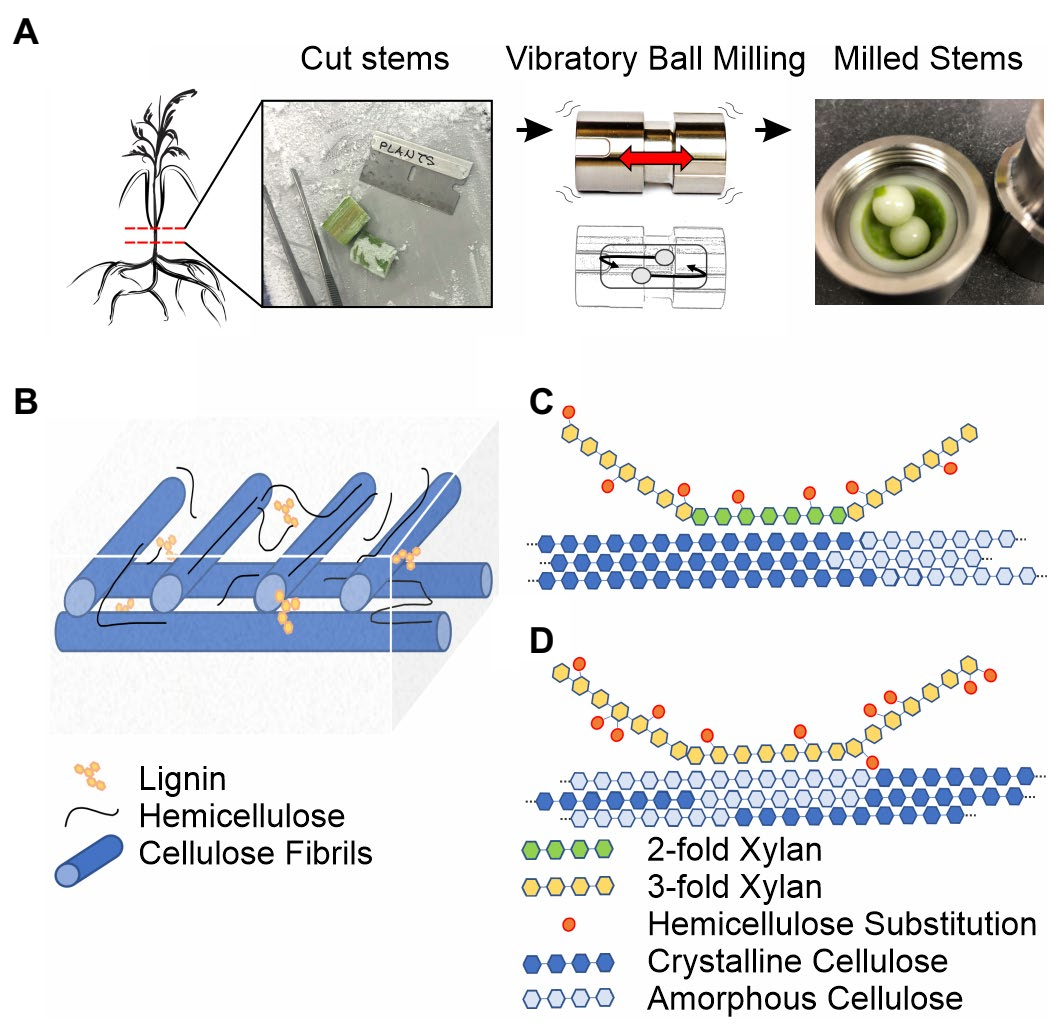
Ball milling approach and overview of the secondary plant cell wall. **(A)** ^13^C-enriched plant stems are harvested, frozen in liquid nitrogen, sectioned on a dry ice cooled surface, and subjected to vibrational ball milling using a zirconium chamber and balls. **(B)** The macroscopic organization of the secondary plant cell wall based on the studies reviewed in Marriot et al. (2016) and Zhang et al. (2021a). Cellulose fibrils are blue, lignin interspersed in the plant cell wall matrix and on carbohydrate surfaces is yellow, and unbound and cellulose-associated hemicellulose is black. **(C)** Solid-state NMR-based model for hemicellulose-cellulose interactions in eudicot Arabidopsis based on Simmons et al. (2016). **(D)** Solid-state NMR-based model for hemicellulose-cellulose interactions in monocot sorghum based on Gao et al. (2020). A major pattern of xylan substitutions is represented as orange ovals in **(C,D)**. Even patterning of glucuronic acid dictates 2-fold xylan associates with crystalline cellulose in eudicots (Simmons et al., 2016; Grantham et al., 2017). The irregular L-arabinosyl xylan substitutions in monocots allow 3-fold xylan and amorphous cellulose interactions (Gao et al., 2020).

Mechanical processing is commonly used to reduce biomass particle size to increase solvent accessibility and polymer solubilization (Zhao et al., 2016; Zhai et al., 2019). Lab-scale vibratory ball milling achieves this goal by rapidly vibrating a chamber containing lignocellulosic biomass with either steel or zirconium balls. An overview of this process is provided in Figure 1A. However, milling leading to recalcitrance is frequently reported during lignocellulosic biomass conversion efforts, impeding the efficiency of subsequent processing and separation steps (Tolbert et al., 2014; Li et al., 2016; Wang et al., 2021). Milling-induced recalcitrance could be due to the production of reactive lignin species (Zhao et al., 2016) promoting aberrant hemicellulose-lignin crosslinks (Terrett and Dupree, 2019) as the lignin recondenses (Li et al., 2016; Kang et al., 2019). Additionally, increased amorphous cellulose content (Ling et al., 2019) could induce reorganization of hemicellulose-cellulose contacts due to their multiple modes of interaction (Simmons et al., 2016; Gao et al., 2020). Importantly, past studies on the plant cell wall structure have used mechanical milling to prepare samples for analysis, so the outcomes may be influenced by non-native interactions and contacts between these polymers (Foston et al., 2012; Park and Cosgrove, 2012; Zhao et al., 2016; Li and Takkellapati, 2018; Ishak et al., 2020).

Recent advances in the incorporation of ^13^C isotopes into living plant tissues allow characterization of the native plant cell wall structure at unprecedented resolution using solid-state Nuclear Magnetic Resonance (NMR; Dick-Pérez et al., 2011; Gao and Mortimer, 2020). Current efforts reveal significant diversity in the organization of plant cell wall polymers across plant species and tissue types (Dick-Pérez et al., 2011; Dupree et al., 2015; Simmons et al., 2016; Kang et al., 2019; Terrett et al., 2019; Gao et al., 2020). After summarizing the current experimental understanding of the plant cell wall architecture with emphasis on solid-state NMR methods, we illustrate the potential of solid-state NMR measurements to assess molecular changes during mechanical preprocessing using *Sorghum bicolor* (sorghum) biomass as an example. The future use of solid-state NMR-based methods to monitor biomass structure during deconstruction will provide an understanding of the molecular mechanisms of recalcitrance, guiding the development of rationally designed deconstruction and crop engineering approaches to enable efficient production of chemical precursors from plants.

## Chemical Composition and Organization of the Secondary Plant Cell Wall

Complexity and insolubility are major barriers for *in situ* characterization of the native plant cell wall structure (Agarwal, 2019; Zhao et al., 2020). The chemical composition of the secondary plant cell wall has been primarily defined using liquid chromatography (Sluiter et al., 2008), mass spectrometry (MS) (Jung et al., 2012; Qi and Volmer, 2019; Zhang et al., 2021b), and solution-state NMR measurements on solvent-extracted polymers (Kim and Ralph, 2010; Foston et al., 2016; Ding et al., 2019) and solid-state NMR (Dick-Pérez et al., 2011; Zhao et al., 2020), vibrational spectroscopy (Agarwal, 2019), and X-ray diffraction (Nishiyama et al., 2002) measurements on native (intact) plant tissues. To help the reader appreciate the potential for polymer reorganization during processing, we present an overview of the current understanding of the secondary plant cell wall chemical composition and architecture.

Figures 1B–D provide an overview of the major polymers present in the secondary plant cell wall and the current understanding of their general macroscopic organization. Interspersed cellulose fibrils provide an immobilized framework for a plant cell wall matrix containing water, soluble proteins, lignin, and hemicellulose polymers (Marriot et al., 2016; Zhang et al., 2021a). The individual cellulose fibrils are built from linear β-(1,4)-glucan polymers composed of crystalline and amorphous arrangements, which differ primarily in their water content and hydrogen bonding patterns, the precise details of which are still under investigation (Song et al., 2020). Hemicellulose polymers both rigidly associate with cellulose fibrils and extend into the matrix environment where they exhibit significant molecular motion and can interact with lignin polymers (Scheller and Ulvskov, 2010; Li et al., 2016; Ding et al., 2019; Kang et al., 2019). Differences across monocot and eudicot plant species have been observed, such as variable substitution patterns on hemicellulose (Simmons et al., 2016; Grantham et al., 2017; Zhang et al., 2019; Gao et al., 2020) and variable hemicellulose-lignin contacts (Scheller and Ulvskov, 2010; Terrett and Dupree, 2019).

The details of lignin structure are particularly challenging to analyze due to its high heterogeneity and mobility, so partial extraction of the polymer is often necessary to assess it (Foston et al., 2012; Li and Takkellapati, 2018; Zhao et al., 2020; Zhang et al., 2021b). Lignin is a polyphenolic network formed from the oxidative cross-linking of the monolignols *p*-coumaryl alcohol (H), coniferyl alcohol (G), and sinapyl alcohol (S) (Li and Takkellapati, 2018). Lignin exists within the plant cell matrix, interfacing with both hemicellulose and cellulose (Foston et al., 2012, Figure 1B). Solution-state NMR in combination with MS, which relies on swelling ball-milled plant cell walls with deuterated solvents, provides detailed information on the types, functionalization, abundance of lignin linkages present, and linkage size (Kim and Ralph, 2010; Pu et al., 2011; Balakshin and Capanema, 2015; Foston et al., 2016; Bergs et al., 2020; Hyväkkö et al., 2020; Zhang et al., 2021b). However, due to the necessity for drying, mechanical treatment, dissolution, or solvent extraction in these techniques, they do not report on recalcitrance in the intact (native) secondary plant cell wall.

## Advances Toward an Accurate 3D Model for the Native Plant Cell Wall Using Solid-State NMR

Like MRI, NMR is a non-invasive way to probe the chemical environment in tissues. Unlike MRI, NMR is inherently an atomic resolution technique, as the observed signals derive from nuclear spin magnetic moments located at precise locations in the molecules under study. In contrast to solution-state NMR, which requires solubilization of the sample, solid-state NMR methods allow for analysis of intact plant tissues. Here, we discuss how solid-state NMR and access to cost effective ^13^C labeling has contributed to our understanding of plant cell wall structure.

The requirement of NMR-active ^13^C isotopes was a major hurdle for characterizations of native plant cell wall structure. ^13^C has a low natural abundance (~1%) and the cost of early efforts at isotope incorporation restricted their use. Relatively low (~11%) ^13^C enrichment enabled early studies on hardwood (Lesage et al., 1999). The ability to detect the relative populations of rigid polymers (e.g., cellulose and a fraction of hemicellulose) was then applied to samples of pure cellulose and heterogeneous assemblies containing cellulose (VanderHart and Atalla, 1984; Newman and Hemmingson, 1995; Lesage et al., 1997; Wickholm et al., 1998; Bootten et al., 2004; Fayon et al., 2005).

While early X-ray diffraction studies demonstrated the crystalline nature of cellulose in plant cell walls (Hauser, 1929; Sisson, 1935; Ward, 1950; Gardner and Blackwell, 1974; Sarko and Muggli, 1974), solid-state NMR measurements provided more detail, such as the pattern of hydrogen bond interactions responsible for the macroscopic shape of *in situ* cellulose fibers (VanderHart and Atalla, 1984; Nishiyama et al., 2002). A set of 1D cross polarization (CP) measurements have been successfully applied to crystalline cellulose in birch and spruce biomass and offer the possibility of detecting exterior and interior cellulose components in macroscopic cellulose fibers (Wickholm et al., 1998; Fernandes et al., 2011). These straightforward 1D experiments were also useful for characterizing amorphous cellulose after ionic liquid processing of crystalline cellulose fibrils (Mori et al., 2012). The 2D Incredible Natural Abundance Double Quantum Transfer Experiment (INADEQUATE) method reports on directly bonded carbon atoms within polymers and has been useful for probing rigid structure, for example, resolving C2, C3, and C5 signals of cellulose in *Populus euramericana* hardwood samples (Lesage et al., 1999) and characterizing the structure of amorphous cellulose (Mori et al., 2012).

A major breakthrough occurred when a highly efficient method of ^13^C incorporation (^13^C glucose feeding to cultured plant cells) was coupled with multi-dimensional solid-state NMR to investigate the primary plant cell wall structure (Dick-Pérez et al., 2011). This series of studies provided both a compositional and architectural description of the primary plant cell wall (Dick-Pérez et al., 2011; Wang et al., 2012, 2013, 2014, 2015; White et al., 2014; Phyo et al., 2017, 2018; Zhao et al., 2020). Hemicellulose-cellulose interactions were found to be much less prevalent in the primary plant cell wall than suggested by earlier models based on solvent-extracted hemicellulose (Keegstra et al., 1973; Talmadge et al., 1973; Pauly et al., 1999; Dick-Pérez et al., 2011; White et al., 2014; Phyo et al., 2017). Furthermore, it was also revealed that the hemicellulose xyloglucan interacts mainly with the flat surfaces of crystalline cellulose fibers (Dick-Pérez et al., 2011; White et al., 2014), expanding on the idea of xyloglucan associating, cross-linking, and embedding into cellulose fibrils (Bootten et al., 2004). Semiquantitative distance measurements recorded with the Proton Driven Spin Diffusion (PDSD) experiments substantiated the organization of cellulose, xyloglucan, and pectin in primary cell walls of both monocot and eudicot cell species (Wang et al., 2012, 2014; White et al., 2014). These advances in ^13^C enrichment have shaped our understanding of the primary plant cell wall architecture (Wang and Hong, 2016).

The strategy of ^13^C glucose feeding is not suitable to the study of the secondary plant cell wall because the plants need to be grown to relative maturity, which is prohibitively expensive and complicated by respiration-dependent glucose synthesis. The development of inexpensive growth chambers, utilizing ^13^C enriched carbon dioxide as the sole carbon source, which support the growth of plants throughout their lifecycle, enabled the efficient incorporation of ^13^C isotopes (>90%) into plant tissues (Chen et al., 2011; Dupree et al., 2015; Gao and Mortimer, 2020). Use of these growth chambers revealed significant differences in the dominant hemicellulose-cellulose contacts in different plant species (Simmons et al., 2016; Terrett et al., 2019; Gao et al., 2020). For example, in eudicot *Arabidopsis thaliana* (Arabidopsis), 2-fold screw conformations of hemicellulose, dictated by even patterns of substitution on xylan, enable a close association with crystalline cellulose (Simmons et al., 2016; Grantham et al., 2017, Figure 1C). In contrast, in monocot sorghum, the high degree and irregularity of arabinose substitution patterns on xylan dictate a 3-fold screw conformation, enabling the association with amorphous cellulose (Gao et al., 2020, Figure 1D). Additionally, in softwoods, cellulose fibrils can be tethered by both xylan and mannan hemicellulose, increasing the strength of the plant cell wall (Terrett et al., 2019). For the sorghum case, limitations in biochemical techniques prevent the analysis of carbohydrate substitutions on xylan and the solid-state NMR measurements therefore describe the xylan-cellulose interaction that is otherwise unobtainable using other methods (Gao et al., 2020, Figure 1D).

Carbon dioxide ^13^C labeling and new applications of advanced solid-state NMR techniques have helped elucidate the structure of lignin in the secondary plant cell wall. Signal enhancement by dynamic nuclear polarization (DNP) demonstrated lignin directly bridges hemicellulose polymers that interact strongly with cellulose fibers in uniformly labeled switchgrass, highlighting the role of lignin in supporting the 3D organization of hemicellulose and cellulose (Kang et al., 2019). However, effective penetration of the DNP reagent into the plant cell wall for this signal enhancement required 15–20 min of milling (Kang et al., 2019), which could perturb native lignin structure. Direct polarization experiments utilizing PDSD (Addison et al., 2020) performed on ^13^C enriched poplar stems highlight a potential avenue to probe lignin contacts and spatial proximities through selective excitation and magnetization transfer from lignin to other polymers, which provide support for the putative organization of lignin in switchgrass (Kang et al., 2019) and Arabidopsis (Dupree et al., 2015). Further development of selective excitation and other solid-state NMR methods to probe biomass with minimal sample manipulation have the potential to provide a more complete picture of the secondary plant cell wall structure and how established sample preparation methods influence that structure.

Although a wide variety of solid-state NMR methods can be applied to highly ^13^C enriched plant tissues, two methods provide rapid and straightforward characterization of the polymer organization in them. First, the INADEQUATE approach provides an avenue for the characterization of the polymers present within a secondary cell wall sample at relatively high resolution and can distinguish at least three populations of amorphous and crystalline cellulose, in addition to three populations of xylan (Simmons et al., 2016; Gao et al., 2020). Second, ^13^C-^13^C recoupling methods, such as PDSD and Dipolar Assisted Rotational Resonance (DARR), report on the spatial proximity of cellulose, hemicellulose, and lignin (Simmons et al., 2016; Grantham et al., 2017; Kang et al., 2019; Terrett et al., 2019; Gao et al., 2020). These experiments provide a more complete picture of contacts between the polymers in the plant cell wall then has ever been obtainable before. For example, the differences hemicellulose-cellulose interactions (Busse-Wicher et al., 2016; Gao et al., 2020) that likely underlie the recalcitrance observed in deconstruction efforts can direct plant selection the biofuel and biomaterials industry.

## Monitoring Secondary Plant Cell Wall Reorganization During Ball Milling

Examining the reorganization of the secondary plant cell wall polymers due to mechanical conversion is important for the development of the plant cell wall model and effective utilization of biomass without recalcitrance (Marriott et al., 2016; Zhao et al., 2020; Zhang et al., 2021a). Solid-state NMR measurements on commercial cotton balls subjected to 15–120 min of vibrational ball milling readily show the conversion of crystalline to amorphous cellulose (Ling et al., 2019). The results are consistent with X-ray diffraction and vibrational spectroscopy measurements that show loss of crystallinity within the cellulose fibers of up to 60% during this same time period (Ling et al., 2019). However, relatively little work has been done on monitoring the native plant cell wall during the milling process. Conversion techniques commonly utilize mechanical milling for times typically as short as 2 min and can exceed 4 h (Kim and Ralph, 2010). As a result, these experiments report on cell wall structure after reorganization of the plant cell wall polymers occurs during mechanical preprocessing. For example, solid-state NMR measurements on maize biomass after mechanical and solvent processing methods support lignin association with the surface of hemicellulose coated cellulose fibers in the cell wall (Foston et al., 2012), a different result than those obtained from recent solid-state NMR measurements on less processed grass and other plant species biomass (Kang et al., 2019; Terrett et al., 2019; Gao et al., 2020). However, these experiments used different sample preparation methods and are from different species, so further investigation is required to make conclusive and precise statements about the influence of these processing methods on plant cell wall structure.

**Figure 2:**
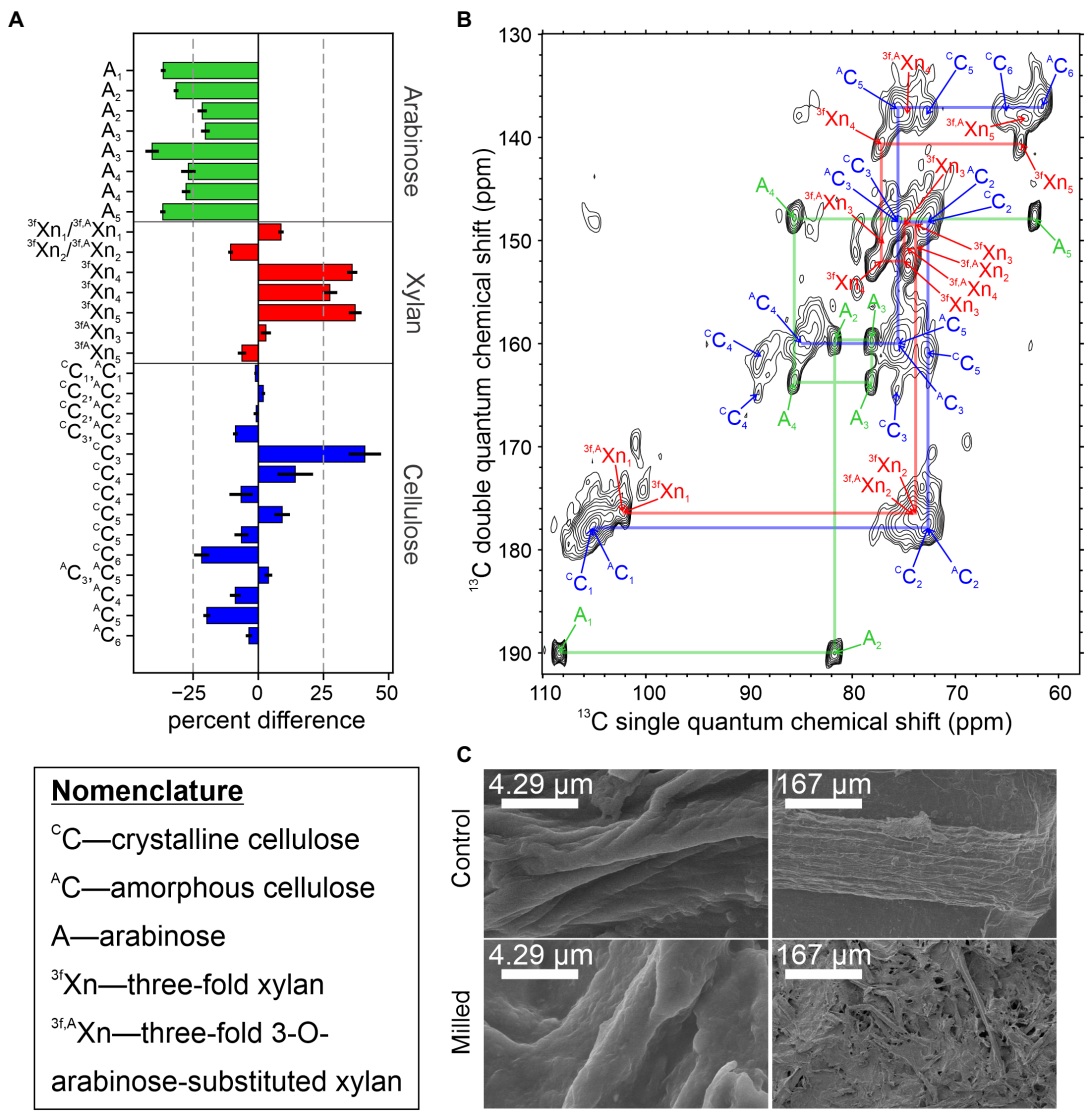
Changes in the rigid structure of sorghum stem tissue after 2 min of ball milling. **(A)** Percent difference in integrated signal intensities from the 2 min ball-milled sample, relative to the unprocessed control sample. Due to the nature of the CP-INADEQUATE experiment, two signals are observed for most sites. Severely overlapping signals were omitted from this analysis. Error bars derive from the root-mean-squared noise in the spectra. **(B)** The CP-INADEQUATE spectrum of the unprocessed control sample. Semi-transparent lines are drawn to show the connections between signals within amorphous cellulose (blue), 3-fold xylan (red), and arabinosyl units from xylan (green). The spectrum was recorded at a ^1^H frequency of 500 MHz with 10 kHz magic angle spinning and high power ^1^H decoupling. **(C)** Field emission SEM images of the 2 min ball-milled and control samples. For all experiments, sorghum Tx430 was grown to maturity in a custom ^13^CO_2_ chamber as previously described (Gao and Mortimer, 2020), and the material used here had at least 92% ^13^C incorporation. Ball milling was adapted from Kim and Ralph (2010), INADEQUATE experiments were performed according to Gao et al. (2020), and samples prepared for FE-SEM and imaged according to Zheng et al. (2020). NMR data were processed in NMRPipe (Delaglio et al., 1995) and plotted in Sparky (Lee et al., 2015).

Currently, we are examining the structural rearrangements that occur in native secondary plant cell walls during mechanical preprocessing using ^13^C sorghum stems subjected to ball milling by solid-state NMR. Milling of stem tissue at 30 Hz for 2 min was selected to allow direct comparison to DMSO swelling studies employing the same milling time (Kim and Ralph, 2010). Figure 2A shows the percent difference in integrated signal intensities from 2D CP-INADEQUATE spectra of the ball milled sample and unprocessed control samples, normalized to minimize the difference in the integrals of the C1-C2 signals of cellulose. Samples were obtained from plants flash frozen in liquid nitrogen after harvest and sectioned on a dry ice cooled surface (Figure 1A). Control experiments show that a cycle of flash freezing does not alter the solid-state NMR spectra of our samples, at least beyond any influence of the initial flash freezing process. Figure 2B shows the polysaccharide region of the control sample spectrum. Signals in these spectra arise from highly immobilized polymers in the secondary plant cell wall (Lesage et al., 1997, 1999). Signal intensities from arabinose all significantly decrease by at least 30% after ball milling while 3-fold xylan C4 and C5 signal intensities increase. The signals from other carbons in xylan, arabinose substituted xylan, and the amorphous and crystalline cellulose intensities do not show a clear trend. Interpretation of these signals is less straightforward due to resonance overlap for these carbohydrates (Gao et al., 2020). The field emission scanning electron microscopy (FE-SEM) images of the control and milled samples in Figure 2C show the macroscopic structure of the plant biomass is lost even after 2 min of ball milling, yet at higher magnification, the individual fibers within the sample are largely similar, with a slightly rougher texture. Although more sophisticated analyses and a wider variety of NMR experiments are possible, this simple example highlights the ability of solid-state NMR to detect structural changes during lignocellulosic biomass conversion.

Although solid-state NMR has the huge advantage of being able to characterize molecular structure of plant cell wall polymers within intact plant material, there are careful considerations to be aware of when planning for these experiments. The time required for 2D solid-state NMR can range from a few hours to almost a day, therefore requiring a few days of experimental time for an extensive set of spectra to be recorded. In our laboratory, we minimize the potential for sample degradation to influence the results of long 2D experiments by interweaving 1D control measurements into the data acquisition. Experiments can be stopped once significant changes in these 1D spectra are observed. Since multiple samples can be obtained from a single plant, dividing acquisition of different data sets between several identical samples is not a problem. Furthermore, the requirement of ^13^C isotope labeled tissue limits the range of plant types and processing methods that can be investigated. We see a bright outlook in this regard with DNP technology, where the future development of non-invasive polarization agents can permit high signal-to-noise data to be obtained from samples with natural abundance ^13^C isotope distributions (Wang et al., 2013; Fernando et al., 2020).

## Conclusion and Outlook

Efficient ^13^C labeling of living plants has opened the door for understanding how biopolymer organization within the plant cell wall confers biomass recalcitrance. Since solid-state NMR does not require solubilization of the plant cell wall, access to ^13^C labeled plant material allows for the native secondary plant cell wall architecture to be tracked during biomass conversion at high resolution. Here, we present a method for quantitative comparison using normalized integration of 2D spectra before and after milling for the refocused CP-INADEQUATE. Although initial results clearly indicate a >30% decrease in arabinose substitutions of xylan, other results remain inconclusive. Future experiments will explore, for example, structural changes in rigid cellulose and the mobile components of hemicellulose and lignin. Contrasts between tissue types containing more or less secondary plant cell wall (e.g., comparing stem and leaf tissue) will also aid in assessing lignin associated recalcitrance. Genetic engineering efforts based on a molecular understanding of recalcitrance will surely also prove invaluable (Carpita and McCann, 2020). In conclusion, recalcitrance remains to be defined within the 3D structure of the plant cell wall and solid-state NMR will be an invaluable tool to investigate native secondary plant cell wall structure and for monitoring structural changes due to genetic engineering and biomass conversion approaches.

## Data Availability Statement

The raw data supporting the conclusions of this article will be made available by the authors upon request, without undue reservation.

## Author Contributions

CRM and DTM recorded and interpreted NMR spectra. CRM recorded the FE-SEM images. YG and JCM produced the ^13^C labeled sorghum plants. CRM, YG, JCM, and DTM wrote the manuscript. All authors contributed to the article and approved the submitted version.

## Funding

This work was made possible by generous start-up funding from the University of California, Davis. The NMR experiments made use of the University of California, Davis NMR Campus Core Facility and Keck Spectral Imaging Facility with funding from the National Science Foundation through NSF EAR0213546. Part of this work was conducted through the DOE Joint BioEnergy Institute (http://www.jbei.org) supported by the U. S. Department of Energy, Office of Science, Office of Biological and Environmental Research, through contract DE-AC02-05CH11231 between Lawrence Berkeley National Laboratory and the U. S. Department of Energy.

## Conflict of Interest

The authors declare that the research was conducted in the absence of any commercial or financial relationships that could be construed as a potential conflict of interest.

## Publisher’s Note

All claims expressed in this article are solely those of the authors and do not necessarily represent those of their affiliated organizations, or those of the publisher, the editors and the reviewers. Any product that may be evaluated in this article, or claim that may be made by its manufacturer, is not guaranteed or endorsed by the publisher.

## Abbreviations

MS: Mass Spectrometry
CP: Cross Polarization
INADEQUATE: Incredible Natural Abundance Double Quantum Transfer Experiment
NMR: Nuclear Magnetic Resonance
PDSD: Proton Driven Spin Diffusion
DARR: Dipolar Assisted Rotational Resonance
FE-SEM: Field Emission Scanning Electron Microscopy
